# Correlation between degenerative spine disease and bone marrow density: a retrospective investigation

**DOI:** 10.1186/s12880-016-0123-2

**Published:** 2016-02-24

**Authors:** Astrid Ellen Grams, Rafael Rehwald, Alexander Bartsch, Sarah Honold, Christian Franz Freyschlag, Michael Knoflach, Elke Ruth Gizewski, Bernhard Glodny

**Affiliations:** Department of Neuroradiology, Medical University of Innsbruck, Anichstraße 35, A-6020 Innsbruck, Austria; Department of Radiology, Medical University of Innsbruck, Anichstraße 35, A-6020 Innsbruck, Austria; Department of Neurosurgery, Medical University of Innsbruck, Anichstraße 35, A-6020 Innsbruck, Austria; Department of Neurology, Medical University of Innsbruck, Anichstraße 35, A-6020 Innsbruck, Austria

**Keywords:** Muskoskeletal imaging, Quantitative computed tomography, Degenerative spine disease, Bone marrow density, Osteopenia, Osteoporosis

## Abstract

**Background:**

Spondylosis leads to an overestimation of bone mineral density (BMD) with dual-energy x-ray absorptiometry (DXA) but not with quantitative computed tomography (QCT). The correlation between degenerative changes of the spine and QCT-BMD was therefore investigated for the first time.

**Methods:**

One hundred thirty-four patients (66 female and 68 male) with a mean age of 49.0 ± 14.6 years (range: 19–88 years) who received a CT scan and QCT-BMD measurements of spine and hip were evaluated retrospectively. The occurrence and severity of spondylosis, osteochondrosis, and spondylarthrosis and the height of the vertebral bodies were assessed.

**Results:**

A negative correlation was found between spinal BMD and number of spondylophytes (*ρ* = −0.35; *p* < 0.01), disc heights (*r* = −0.33; *p* < 0.01), number of discal air inclusions (*ρ* = −0.34; *p* < 0.01), the number of Schmorl nodules (*ρ* = −0.25; *p* < 0.01), the number (*ρ* = −0.219; *p* < 0.05) and the degree (*ρ* = −0.220; *p* < 0.05) of spondylarthrosis. Spinal and hip BMD correlated moderately, but the latter did not correlate with degenerative changes of the spine. In linear regression models age, osteochondrosis and spondylarthrosis were factors influencing spinal BMD.

**Conclusion:**

Degenerative spinal changes may be associated with reduced regional spinal mineralization. This knowledge could lead to a modification of treatment of degenerative spine disease with early treatment of osteopenia to prevent secondary fractures.

## Background

Data from prior studies are inconclusive with respect to the relationship between bone mineral density (BMD) and degenerative changes of the spine. In the few studies conducted to date, dual-energy x-ray absorptiometry (DXA) has been compared with either x-ray or magnetic resonance imaging studies. In some cases there was a positive [1, 2], in others a negative [3, 4] or no relationship [5] with each other. It is known that the presence of spondylophytes, one component of degenerative spine disease, impairs DXA-BMD measurements in the spine; BMD is systematically overestimated [6]. This overestimation from adjacent dense structures can be avoided by applying quantitative computed tomography (QCT) [7]. Other advantages of this method are that conventional CT scanners can be used, cortical and trabecular BMD can be differentiated, and size-independent, volumetric BMD data can be gained [8]. This leads to advantages in the assessment of changes in bone density over time [8]. Moreover, the bone density can also be calculated from examinations that are not conducted for this purpose, for example from cardiac computed tomographies [9]. The disadvantages are the greater radiation dose – at least in measurements of the spine – for QCT compared with DXA, the lack of data about the prognostic value with respect to future fractures, and the lack of applicability of the WHO criteria regarding the T-score for diagnosing osteoporosis [8]. Although QCT-BMD values, unlike DXA measurements, are not influenced by adjacent dense structures, the method has not yet been used to examine potential relationships between BMD and degenerative changes of the spine itself.

Therefore, the aim of this study was to investigate the possible relationship between different measurable morphological changes of degenerative spine disease detected by CT and the BMD measured by QCT from the same data set.

## Methods

In this study, 134 patients (66 female and 68 male) with a mean age of 49.0 ± 14.6 years (range: 19–88 years) who received a CT scan and a quantitative computed tomography BMD measurement of the thoracolumbar spine and the hip were evaluated retrospectively. The study was approved by the Ethics Committee of the Medical University of Innsbruck (Reference number: AN2014-0106335/4.19).

Patients were selected from a cohort of 1249 patients who received a CT scan of the trunk between the years 2007 and 2014 as well QCT-BMD measurements of the thoracolumbar spine and the hip. The patients of this population were examined by CT scan due to chronic back pain. Patients with ankylosing spondylosis, other inflammatory diseases such as psoriasis, fractures, plasmacytoma, multiple myeloma, monoclonal gammopathy, and suspected tumor disease (*n* = 1114) were excluded from the study. None of the selected patients suffered from any of the aforementioned diseases or had any visible bony changes such as fractures, except for degenerative spine disease.

The CT scans were acquired on a LightSpeed 16 or a LightSpeed VCT scanner (General Electric, GE Healthcare, Chalfont St Giles, Buckinghamshire, UK), the latter since 2012. A tube voltage of 120 kV was used in all scans, coupled with automatic adaptation of the current to a predetermined noise factor. Data were acquired in 2.5 mm slice thicknesses. There were always 0.625-mm slice thicknesses available in a bone algorithm, as well coronal and sagittal reconstructions of a slice thickness and a slice interval of 3.0 mm.

**Fig. 1:**
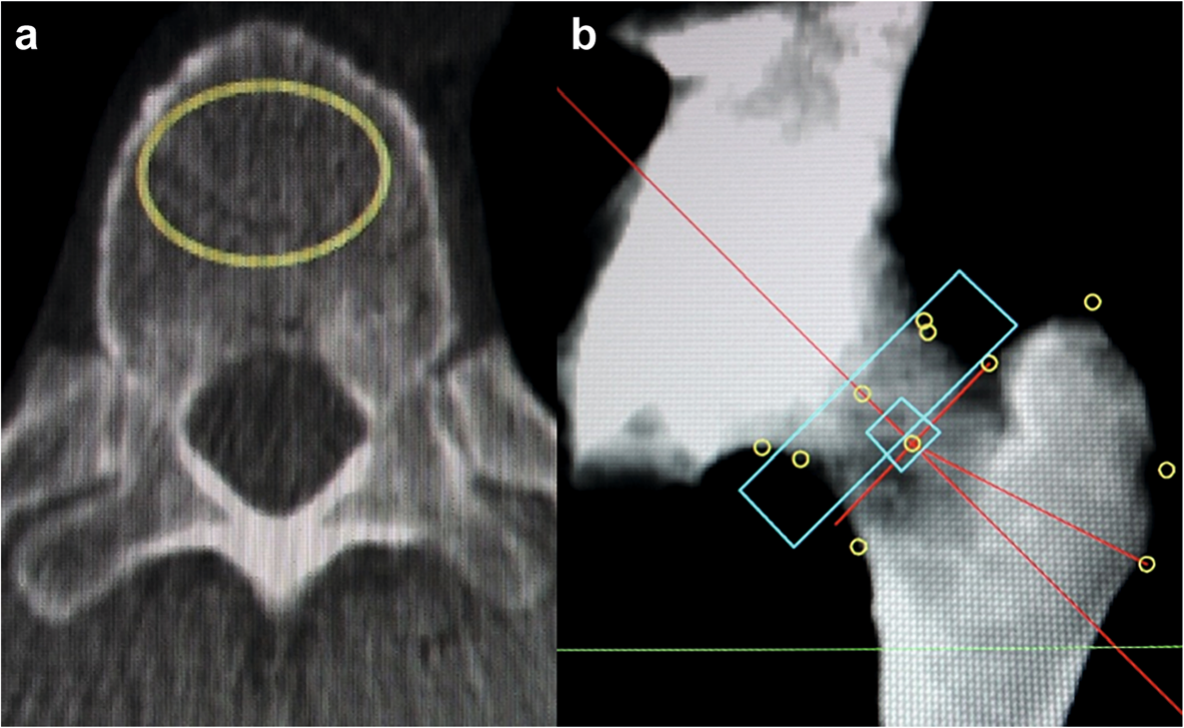
QCT BMD measurements. Examples for quantitative computed tomography bone marrow density measurements in the spine (**a**) and the femur (**b**)

Measurements were made by two pairs of observers (AB, SH; AEG, BG). The subjects were selected by two experienced, board-certified radiologists (AEG, BG). To evaluate spondylosis, the number and type of spondylophytes were counted in the entire spine and classified as “marginal spondylophytes” or as “hyperostotic spondylophytes” [10] (Fig. 1a, b). For the assessment of osteochondrosis, the heights of the discs were measured and the number of air inclusions in the discs was counted, as well as the number of Schmorl nodules and sclerotic endplates between the 12^th^ thoracic and the first sacral vertebra. To evaluate spondylarthrosis, the number and severity of degenerative changes of the facet joints between the 12^th^ thoracic and the first sacral vertebra were noted according to a previously published method [11] (Fig. 1c, d), and classified as “normal joint”, “narrowed joint space = stage 1”, “narrowed joint space and sclerosis or hypertrophy of the facet = stage 2” and “narrowed joint space, sclerosis and spondylophytes = stage 3”. Additionally the heights of the vertebral bodies between the 12^th^ thoracic and the first sacral vertebra were measured.

**Fig. 2:**
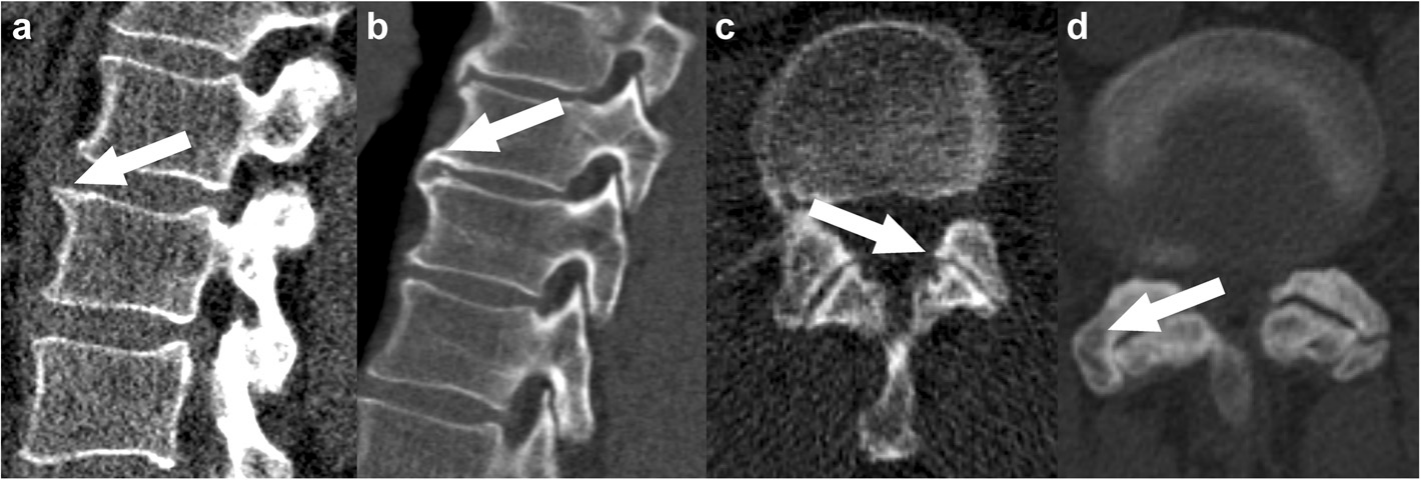
Examples for degenerative spine disease. Examples of marginal (**a**) and hyperostotic (**b**) spondylophytes, as well as spondylarthrosis grade 1 (**c**) and 3 (**d**); arrows are pointing at the pathologies

The QCT-BMD was measured in at least three segments of the thoracolumbar spine between the 11^th^ thoracic and the 3^rd^ lumbar segment (Fig. 2a). To do this, a region of interest was marked in the center of the vertebral body at a distance of 2–3 mm from the cortex [9], in which the trabecular BMD was calculated in mg/cm^3^ of hydroxylapatite. Enostoses, cortical bone, sclerotic zones, and Schmorl nodules were excluded. If this was not possible, an adjacent vertebral body was used. Then the mean of the three vertebral bodies was formed. The practice guidelines of the American College of Radiologists [12] were followed.

Using the femur on one side, the BMD was calculated from the trabecular BMD of the trochanter major, the femoral neck, and the intertrochanteric region (Fig. 2b) in the corresponding ROIs, taking the volume into consideration. For measuring the spinal column and the femur, a Mindways QA phantom (QA Phantom Model 3, Mindways, San Francisco, CA, USA) was used in conjuction with the corresponding software (QCT PRO version 4.2.3, Mindways, San Francisco, CA, USA).

For statistical evaluation, the software Excel (Office 2013, Microsoft, Redmond, WA, USA), GraphPad Prism 6 (GraphPad Software, Inc., La Jolla, CA, USA), and SPSS (IBM SPSS Statistics for Windows, Version 22.0.. IBM Corp, Armonk, NY, USA) were used.

Distributions were tested for normality using the Kolmogorov-Smirnov test. Depending on the result, either the Spearman (ρ) or the Pearson (r) test was used for correlation analyses. Comparisons between three or more groups were made using the Kruskal-Wallis test in combination with Dunn’s post hoc test or a one-way ANOVA together with a Bonferroni correction, as appropriate. Finally, different linear regression models were fitted to the target variables “BMD”. This was carried out first including all variables, then using stepwise forward selection technique. A *p* < 0.05 was regarded to be significant.

## Results

Women displayed a mean spinal QCT-BMD of 256.6 ± 41.6 mg/cm^3^, men a mean BMD of 252.56 ± 50.03 mg/cm^3^. A significant negative correlation was found between BMD and age (*r* = −0.62; *p* > 0.01). Mean results for the measured pathologies of the entire population classified by gender are given in Table 1.

**Table 1:** Observed pathologies by gender

Pathology	All patients	Female	Male
Spondylosis deformans			
Spondylophytes spine	10.55 ± 15.48	10.12 ± 14.02	10.97 ± 16.87
Grade 1	7.76 ± 11.78	7.48 ± 10.97	8.03 ± 12.59
Grade 2	2.38 ± 4.39	2.21 ± 3.82	2.54 ± 4.90
Grade 3	0.41 ± 1.41	0.42 ± 1.38	0.40 ± 1.45
Osteochondrosis			
Disc height (mm)	8.91 ± 1.59	8.51 ± 1.4	9.30 ± 1.68
Air inclusions	0.43 ± 0.99	0.52 ± 1.10	0.35 ± 0.88
Schmorl nodules	1.49 ± 2.28	1.48 ± 2.14	1.50 ± 2.42
Endplate sclerosis	0.12 ± 0.49	0.11 ± 0.43	0.13 ± 0.54
Spondylarthrosis			
Highest grade	0.43 ± 0.87	0.56 ± 0.99	0.29 ± 0.71
Average grade	0.09 ± 0.25	0.11 ± 0.27	0.07 ± 0.23
Number	0.61 ± 1.51	0.74 ± 1.67	0.49 ± 1.33
Vertebral body height (mm)	28.14 ± 1.63	27.55 ± 1.54	28.72 ± 1.52

Significant positive correlations (*p* < 0.01 each) were found between patient age and the number of spondylophytes (*r* = 0.53), the disc height (*r* = 0.25), the number of discs showing air inclusions (*ρ* = 0.43), the number of endplates affected by Schmorl nodules (*ρ* = 0.25), and the number of facet joints showing spondylarthrosis (*ρ* = 0.33). A slight, but significant negative correlation was found between age and vertebral body height (r = −0.18; *p* < 0.05).

**Fig. 3:**
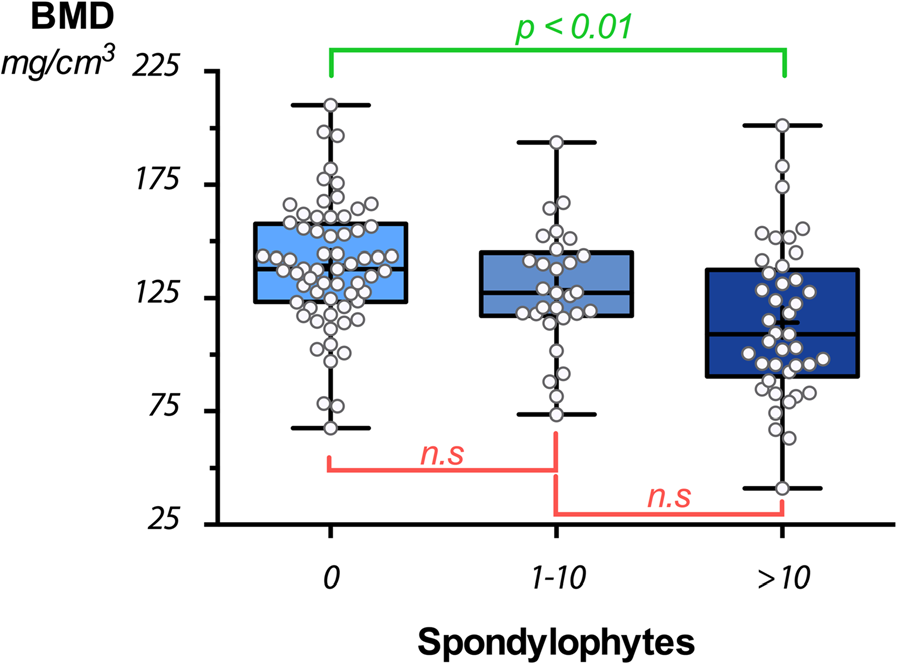
Correlation between BMD and number of spondylophytes. Comparison of the bone marrow density between patients without spondylophytes, patients with >1, but <10 spondylophytes, and patients with >10 spondylophytes. Dunn’s multiple comparisons test reveals significant differences between the group „0“and „ > 10” spondylophytes“ only

**Fig. 4:**
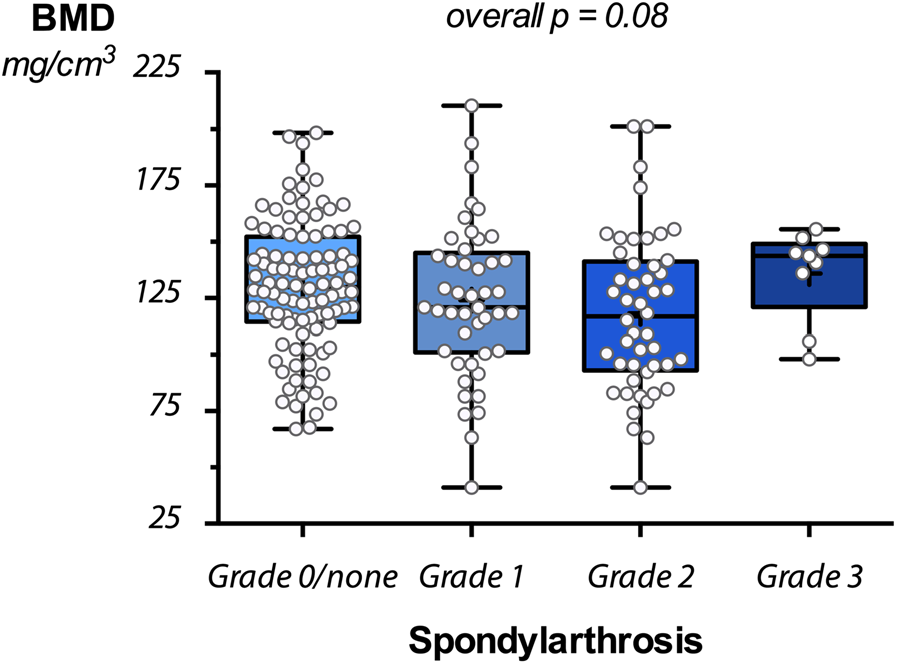
Correlation between BMD and grade of spondylarthrosis. Comparison of the bone marrow density measurements between patients without spondylarthrosis, patients with grade 1, grade 2, and grade 3 spondylarthrosis. The differences were not significant

Spinal BMD was found to be significantly lower in patients with spondylophytes, compared to patients without spondylophytes (Fig. 3), but not in patients with spondylarthrosis compared to patients without spondylarthrosis (Fig. 4). The type of spondylophytes was irrelevant.

There were significantly negative correlations between the number of spondylophytes, disc height, the number of discs showing air inclusions, the number of endplates affected by Schmorl nodules, and the number of facet joints showing spondylarthrosis and the BMD of the spine (Table 2).

**Table 2:** Correlations of degenerative changes of the sine with spinal and hip BMD

Pathology	Normal distribution^a^	Correlation with BMD spine	*p*	Correlation with BMD hip	*p*
Spondylosis deformans					
Spondylophytes spine	no	−0.349	<0.01	−0.077	n.s.
Grade 1	no	−0.341	<0.01	−0.072	n.s.
Grade 2	no	−0.345	<0.01	−0.048	n.s.
Grade 3	no	−0.197	<0.05	0.003	n.s.
Osteochondrosis					
Disc height (mm)	yes	−0.332	<0.01	−0.307	<0.01
Air inclusions	no	−0.341	<0.01	−0.113	n.s.
Schmorl nodules	no	−0.246	<0.01	−0.176	0.046
Endplate sclerosis	no	0.147	0.09	0.085	n.s.
Spondylarthrosis					
Highest grade	no	−0.223	<0.01	−0.080	n.s.
Average grade	no	−0.220	<0.05	−0.077	n.s.
Number	no	−0.219	<0.05	−0.072	n.s.
Vertebral body height (mm)	yes	0.139	0.11	0.008	n.s.

^a^Kolmogorov-Smirnov test, if normal distribution assumed: Pearson correlation coefficient, if not: Spearman correlation (ρ)

BMD of the spine correlated significantly with the BMD of the hip (*r* = 0.45; *p* < 0.01). However, correlations between the BMD of the hip and the degenerative changes of the spine were weaker (Schmorl nodules and disk height), insignificant, or absent (Table 2).

In linear regression models age, osteochondrosis and spondylarthrosis were factors with a negative influence on the spine BMD (Table 3).

**Table 3:** Linear Regression model (stepwise method)

	Beta	Sig.
Dependent: BMD spine		
Patient age (years)	−0.571	<0.001
Osteochondrosis (Disc height)	−0.202	0.002
Spondylarthrosis (Number)	−0.162	0.016

## Discussion

In theory, degenerative intervertebral disc disease leads to adaptive changes of the adjacent bone with decreased density of the trabecular core and increased density of the cortical vertebral walls [13]. In this study, we were able to underline this hypothesis by finding a lower BMD with increasing severity of degenerative changes in the spine. Due to the fact that the number and degree of degenerative changes correlate inversely with the BMD of the spine, but except for osteochondrosis in terms of the number of Schmorl nodules and disc height not with the BMD of the femur, the loss of BMD may be a local epiphenomenon of the degenerative processes.

In an earlier study it was shown that the height of the discs correlates positively with the spinal DXA-BMD in premenopausal women [4]. However, studies investigating spondylophyte formation [1, 14, 15] or disc degeneration [16] found an increasing DXA-BMD with increasing degenerative changes, such as presence of spondylophytes. In view of the present results, showing a negative association of BMD with degenerative spine disease, the studies cited may be interpreted in a new way; a decrease of disc height as an early stage of detectable degeneration does not appear to affect DXA-BMD, whereas more advanced changes such as spondylophytes occurring later in the degenerative process do [6, 17]. The BMD measured using QCT seems to be more sensitive for slight demineralization [8] going along with early degenerative changes. With the progress of the degeneration and volume loss of the intervertebral disc, the affected segment becomes unstable in a later state, and the adjacent ligaments become loose. This instability can lead to abnormal stress, which can result in releasing nitrogen from the tissue due to a transient negative pressure. The visible air inclusions in the discs or joint spaces are a marker for instability, and consequently are accompanied by demineralization as well.

Degenerative spondylophytes, which are formed as a bracing reaction due to instability, are not associated with an increase of DXA-BMD, as has been previously assumed [1, 2], but with a decrease. This can be explained by several effects. Spondylophytes, as densely calcified structures, may lead to the systematic overestimation of DXA-BMD. Moreover, tilting or rotation of the vertebral bodies occurs with advanced degenerative changes, which could result in further overestimation of the adjacent bone mineral density from DXA-BMD due to summation effects [6]. This overestimation can be avoided with QCT as ROIs can be placed in selected areas and therefore cortical or adjacent structures are not included into the area of measurement [7]. Volumetric, size-independent BMD data can be gained, and cortical and trabecular BMD can be differentiated [8].

In agreement with the literature [5], weak or no correlations between hip QCT-BMD and disc degeneration parameters were found, but a significant correlation was found between the QCT-BMDs of the hip and the spine. This can be interpreted to be an indication that the negative correlation between the degenerative changes of the spine and the BMD could be a local phenomenon. However, due to the retrospective design of this study, it is not possible to identify demineralization as a consequence of the progression of degeneration. Only a longitudinal, prospective observational study would be suitable for this.

A basic limitation of the study is the retrospective design. No information about medication was available in the examined population, so the BMD of the spine could have been influenced from unknown intake of bisphosphonates, for example. Although patients with obvious bony manifestations of diseases were excluded, it is conceivable that disease-associated changes such as micrometastases may have influenced these results. Another limitation is that QCT requires a higher radiation dose than other BMD methods. In the investigated study population, BMD was acquired to estimate the risk of a fracture only when a disease with affection of the bone was suspected and not for study reasons. This is why only about 11 % of the entire population was selectable, as they did not feature bony changes due to their suspected disease or they did not suffer from the suspected disease. The last limitation is that estimation errors of absolute BMD values may have occurred using QCT as well. We did not examine possible reasons for such effects. However, as the focus of our work lay on the detection of relative changes of BMD in the context of degenerative processes, systematic estimation errors are supposed to be of minor importance only.

The results of the present study will have to be proven in prospective investigations or in a population who received both DXA-BMD and QCT-BMD, especially due to the results that partially contradicted the earlier DXA studies. More detailed research on a larger patient population would be useful for better understanding the entire process of demineralization along with degenerative alterations.

## Conclusion

The results of the present study show that degenerative changes of the spine, from loss of disc height to formation of spondylophytes, are accompanied by demineralization of the bone. The lack or insignificance of associations between degenerative changes of the spine and the BMD of the femur, and at the same time moderate associations between the BMD of the spine and of the hip itself may be interpreted to be an indication that degenerative changes of the spine could be the cause of local BMD loss. However, this assumption can be proven only in a longitudinal prospective study.

This knowledge may entail modifications of therapy for degenerative spine disease in the future, for example in the earlier initiation of osteoporosis therapy, in order to improve the prevention of serious sequelae for patients with degenerative spine disease.

### Ethics approval

This study was approved by the Ethics Committee of the Medical University of Innsbruck (Reference number: AN2014-0106335/4.19).

Ethikkommission der Medizinischen Universität Innsbruck Innrain 43, A-6020 Innsbruck, Austria.

### Availability of supporting data

Due to statutory provisions regarding data- and privacy protection, the dataset supporting the conclusions of this article is only available upon individual request directed to the corresponding author.

## Abbreviations

ANOVA: analysis of variance
BMD: bone mineral density
CT: computed tomography
DXA: dual-energy x-ray absorptiometry
QCT: quantitative computed tomography
r: Pearson’s r
WHO: World Health Organization
ρ: Spearman rho
